# Distal coronary embolization following acute myocardial infarction increases early infarct size and late left ventricular wall thinning in a porcine model

**DOI:** 10.1186/s12968-015-0197-y

**Published:** 2015-12-01

**Authors:** Reuben M. Thomas, Sang Yup Lim, Beiping Qiang, Azriel B. Osherov, Nilesh R. Ghugre, Hossein Noyan, Xiuling Qi, Rafael Wolff, Michelle Ladouceur-Wodzak, Thomas A. Berk, Jagdish Butany, Mansoor Husain, Graham A. Wright, Bradley H. Strauss

**Affiliations:** Schulich Heart Centre, Sunnybrook Health Sciences Center, 2075 Bayview Avenue, Room D-406, Toronto, ON M4N 3M5 Canada; Physical Sciences Platform, Sunnybrook Research Institute, Sunnybrook Health Sciences Centre, Toronto, Canada; Toronto General Research Institute, Toronto, Canada; Department of Pathology, University Health Network, Toronto, Canada; University of Toronto, Toronto, Canada; Korea University Ansan Hospital, Ansan, Korea; Barzilai Medical Centre, Ashkelon, Israel

**Keywords:** Cardiovascular magnetic resonance, Myocardial infarction, Angioplasty, No reflow

## Abstract

**Background:**

Distal coronary embolization (DCE) of thrombotic material occurs frequently during percutaneous interventions for acute myocardial infarction and can alter coronary flow grades. The significance of DCE on infarct size and myocardial function remains unsettled. The aims of this study were to evaluate the effects of DCE sufficient to cause no-reflow on infarct size, cardiac function and ventricular remodeling in a porcine acute myocardial infarction model.

**Methods and results:**

Female Yorkshire pigs underwent 60 min balloon occlusion of the left anterior descending coronary artery followed by reperfusion and injection of either microthrombi (prepared from autologous porcine blood) sufficient to cause no-reflow (DCE), or saline (control). Animals were sacrificed at 3 h (*n* = 5), 3 days (*n* = 20) or 6 weeks (*n* = 20) post-AMI. Cardiovascular magnetic resonance (CMR), serum troponin-I, and cardiac gelatinase (MMP) and survival kinase (Akt) activities were assessed. At 3d, DCE increased infarct size (CMR: 18.8 % vs. 14.5 %, *p* = 0.04; serum troponin-I: 13.3 vs. 6.9 ng/uL, *p* < 0.05) and MMP-2 activity levels (0.81 vs. 0.49, *p* = 0.002), with reduced activation of Akt (0.06 versus 0.26, *p* = 0.02). At 6 weeks, there were no differences in infarct size, ventricular volume or ejection fraction between the two groups, although infarct transmurality (70 % vs. 57 %, *p*< 0.04) and ventricular thinning (percent change in mid anteroseptal wall thickness:-25.6 % vs. 0.7 %, *p* = 0.03) were significantly increased in the DCE group.

**Conclusions:**

DCE increased early infarct size, but without affecting later infarct size, cardiac function or ventricular volumes. The significance of the later remodelling changes (ventricular thinning and transmurality) following DCE, possibly due to changes in MMP-2 activity and Akt activation, merits further study.

## Background

Treatment of acutely occluded coronary arteries by percutaneous coronary intervention (PCI) is the preferred modality for patients with acute myocardial infarction (AMI) [1]. However, even after successful reperfusion therapy, reduced myocardial flow with suboptimal perfusion of the myocardium at the tissue level (Thrombolysis in Myocardial Infarction (TIMI) flow grades 0-2) is common, occurring in approximately 20 % of patients at some point during the procedure [2, 3]. In its most extreme form, TIMI-0 or -1 flow, known as reperfusion no-reflow (NR), has been associated with increased infarct size, reduced recovery of ventricular function, and increased mortality [4]. The underlying cause of NR is due to microvascular injury, characterized by damage to the myocardial microvasculature, and microvascular obstruction (MVO), thereby limiting tissue level perfusion [5]. Microvascular injury has been attributed to both the initial ischemic injury and the effects of reperfusion injury [6].

Distal coronary embolization (DCE) of plaque and acute thrombotic material at the arterial occlusion site due to guide wire crossing and/or balloon inflation and stent deployment has been identified as another important cause of microvascular injury [7]. In North America [8], the use of thrombus aspiration catheters as an adjunct to PCI for ST-elevation myocardial infarction is recommended. This recommendation stems from mortality benefits observed in the TAPAS trial [9]. However, inconsistent effects on infarct size and mortality benefits in subsequent clinical studies have raised questions on the routine use of these devices in Primary PCI procedures for AMI [10, 11]. Currently the role of DCE and need for therapy remain controversial.

To date, few pre-clinical studies have addressed the effects of DCE immediately following acute ischemia on microvascular injury, infarct size and myocardial repair processes at early and later time points. Moreover, previous studies have utilized polystyrene microspheres as embolization material, which may not be reflective of the true biological process [12]. In the current study, the objectives were to characterize by cardiovascular magnetic resonance (CMR) the effects of NR on myocardial infarction and subsequent cardiac remodelling changes following DCE of biologically active, blood clot material in a porcine AMI model.

## Methods

### Microthrombi preparation and characterization

The microthrombi used for DCE were derived from autologous porcine blood. Three mL of blood was collected in a standard serum separating tube and the resulting clot was heated to 80–90 °C until completely dry (~2 h). The dried clot was mechanically ground and 5 mg was weighed into Eppendorf tubes, resuspended in sterile saline to a final concentration of 2 mg/ml and injected following 60 min of ischemia.

Microthrombi particle size was determined using a Multisizer™ III Coulter Counter (Beckman Coulter Inc, California, USA) and a 400 μm aperture tube. Five mg of microthrombi was suspended in 300 mL of isoton-II electrolyte solution and particle sizes were calculated over a 20s run. This was replicated three times using DCE samples from eight different animals.

To assess for coagulation effects of microthrombi, porcine blood was collected 1 h post injection of heparin (3000 units) and incubated with increasing amounts of microthrombi for 1 h at room temperature. Measurements of activated clotting time (ACT) were done using a HEMOCHRON Jr. Signature + Whole Blood Microcoagulation System (ITC, Edison, NJ).

### In vivo AMI model

Animal procedures were approved by the Animal Care Committee at Sunnybrook Health Sciences Centre. Female Yorkshire pigs (25–35 kg, Vista Village Farms, Ontario) were sedated using a mixture of ketamine (25 mg/kg) and atropine (0.05 mg/kg) injected I.M and then mechanically ventilated with 1–3 % isoflurane. Buprenorphine (0.05 mg/kg) was administered I.M pre- and post-operatively and Metacam (0.2 mL/kg) PO twice daily for 2 days for pain management. During the baseline procedure, the left main coronary artery was engaged using a 6Fr JL 3.0 guiding catheter (Medtronic, Minneapolis, MN). A Twin-Pass® dual access catheter (Vascular Solutions Inc, Minneapolis, MN) was placed distal to the second diagonal branch of the left anterior descending (LAD) coronary artery. The animals were placed in a 3.0T MRI scanner and imaged as described below.

Following a 4–5 days period of recovery from the baseline procedure, animals were returned to the catheterization laboratory. An angioplasty balloon catheter, 2.0–2.5 mm diameter (Sprinter Legend, Medtronic, Minneapolis, MN), depending on vessel size, was then used to occlude the LAD artery for 60 min, at the same position used in the baseline study. This period of ischemic time was deliberately chosen to cause a moderate size, patchy myocardial infarction. Previous studies from our group have indicated that 90 min ischemia causes a large (20–30 % of LV), transmural infarction with extensive MVO, while 45 min ischemia results in a small or negligible infarct size without MVO [13, 14]. Treatment of arrhythmias with antiarrhythmic drugs (including lidocaine, atropine, epinephrine, and amiodarone), and/or defibrillation during the ischemic or reperfusion period were used as required. Following ischemia, the balloon was deflated and removed, and the coronary flow grade was assessed at angiography. An over-the-wire balloon catheter was then advanced to the same location and inflated. Administration of 2.5 ml of either microthrombi particles suspended in saline (“DCE”) or sterile saline (“control”) was done through the guide wire port of the over-the-wire balloon catheter, followed by a 0.5 ml saline flush. Coronary flow grade was assessed immediately following injection. Microthrombi particle injection was repeated in the DCE group until TIMI-0 or TIMI-I flow was present. The final TIMI flow grade was again assessed at 10 min post injection. No additional microthrombi particles were injected based on the final TIMI flow grade.

**Fig. 1:**
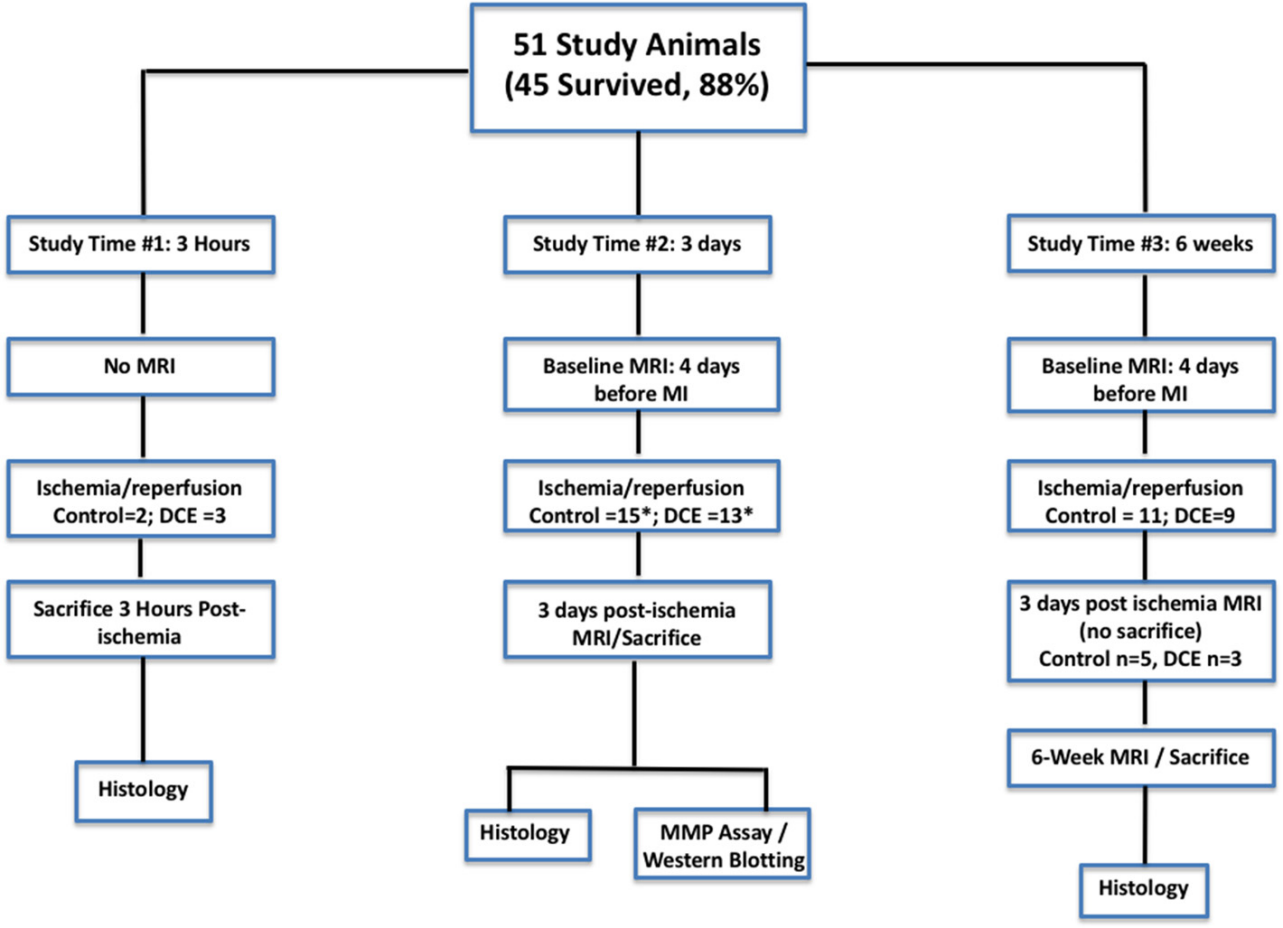
Schematic showing the number of animals utilized in each experimental group. Out of 51 animals, 45 survived to the desired endpoints. *n* = 5 animals were part of study 1 (no MRI, sacrificed 3 h post ischemia); *n* = 20 animals were part of study 2 (sacrificed at 3 days post ischemia); *n* = 20 animals were part of study 3 (sacrificed 6-weeks post ischemia). A subset of animals in study 3 (Control *n*= 5, DCE *n* = 3) were imaged at 3 days post ischemia and included with the results from study 2

Animals were subsequently imaged and sacrificed (overdose of pentobarbital sodium injected IV) at one of three time points: immediate (3 h post procedure (no CMR imaging), *n* = 5 including control = 3 and DCE = 2), early (3d post procedure, *n* = 20, 10 per group) and late (6 weeks post procedure, *n* = 20, control = 11, DCE = 9). The study scheme and sample sizes are indicated in Fig. 1. Troponin-I levels were measured at baseline and at 3d.

### Coronary angiography

Coronary angiograms were performed at five time points in the study: 1) immediately post 60 min balloon inflation, 2) immediately post saline or microthrombi injection, 3) at 10 min post saline or microthrombi injection, 4) at 3d post-ischemia (early group plus eight pigs in the 6 weeks group) and 5) at 6 weeks (late group).

### CMR

CMR was conducted using a 3.0T whole body MR scanner (MR 750, GE Healthcare, Waukesha, WI). Cardiac gating was achieved using a peripheral plethysmograph and an 8 channel cardiac coil was utilized for image acquisition for all CMR scans. CMR was conducted at baseline, at 3d and 6 weeks post-AMI. The objectives of the baseline CMR scan were to determine cardiac function and the myocardial perfusion territory-at-risk at the mid-level of the LAD where the subsequent control or DCE injection would occur. The risk territory was determined by the region of enhanced myocardium following intracoronary injection of dilute gadolinium-DTPA (0.015 mmol/kg Magnevist, Bayer Healthcare) through the Twin-Pass® dual access catheter, as previously described [15]. Perfusion territory images were acquired using a T1-weighted inversion recovery fast gradient echo (IR-FGRE typical parameters: TR/TE = 5.7/2.6, flip angle = 15°, field of view = 24 × 21.6 cm, acquisition matrix = 192 × 160, acquisition gated to diastole). Cardiac function was characterized using a cine steady-state-free-precession sequence (FIESTA, GE Healthcare) with 12 to 15 short-axis slices acquired from apex to base under breath-hold conditions (TR/TE = 4.2/1.7, flip angle = 45°, field of view = 24 × 21.6 cm, acquisition matrix = 224 × 160, eight views per segment, 20 cardiac phases/slice).

At the endpoint scans (3 days or 6 weeks post AMI), cine images were acquired as described above. Gadolinium-DTPA (0.2 mmol/kg Magnevist, Bayer Healthcare) was injected intravenously to determine infarct size and MVO. Late gadolinium enhancement (LGE) imaging (imaging ten minutes following contrast injection) was utilized to characterize infarct volume and persistent MVO (IR-FGRE- typical parameters: TR/TE = 4.1/1.9 ms, flip angle = 15°, field of view = 24 × 21.6 cm, acquisition matrix = 224 × 160, 2 RR intervals, inversion time adjusted between 250 and 300 ms to null signal from normal myocardium, acquisition gated to diastole).

### CMR image analysis

CMR tissue characteristics were quantified using custom MATLAB® scripts (The Mathworks Inc., Natick, MA).

#### Baseline perfusion territory

Volumetric values of the myocardial perfusion territory were quantified from the baseline contrast enhanced images. Myocardial signal intensities that were two standard deviations above mean remote myocardial signal intensities were highlighted.

#### Infarct size

Infarct size was determined using the late gadolinium enhancement (LGE) CMR images and are reported in two ways: 1) percent of the overall left ventricular (LV) mass and 2) percent of the mid LV territory at-risk based on the baseline intracoronary gadolinium-DTPA injections prior to the infarction. The full width at half maximum method [16] was utilized to delineate regions of infarcted myocardium.

#### Microvascular obstruction (MVO)

Early gadolinium enhancement images (EGE), acquired 2 min after intravenous contrast injection, were utilized to characterize the presence or absence of MVO. Persistent MVO was quantified from LGE images acquired 10 min post injection of contrast. Regions of hypo-enhancement within a zone of hyper-enhancement were manually traced and volumes were represented as percent of LV mass.

#### Transmurality

The maximum radial transmurality was calculated for a given myocardial slice as described previously [14], and averaged across all infarcted, or three mid-infarct myocardial slices.

#### Global and regional left ventricular function

Short axis cine images were analyzed using the QMass MR software (Medis, Netherlands). Epicardial and endocardial contours were traced for the end diastolic and end systolic phases, and volumetric data were compiled. Analysis of regional cardiac functional parameters was conducted using the QMass MR software. The posterior junction between the right and left ventricle was prescribed, which allowed for the segmentation of the myocardium based on the American Heart Association 17 segment model [17]. Measurements of end diastolic wall thickness were determined for segment 8 (mid-anteroseptal), which represented the core region of the infarct.

### Tissue processing

Following sacrifice, hearts were excised and sliced into 7–8 short axis sections. Tissue samples from 3d post-AMI hearts were taken from the core-infarct of the mid infarct slice and flash frozen in liquid nitrogen for gelatin zymography and western blotting experiments described below. The remaining slices were fixed in 10 % neutral buffered formalin, and paraffin-embedded sections were stained with hematoxylin and eosin (H&E) and reviewed by an experienced cardiac pathologist (JB).

### Gelatin zymography

Matrix metalloproteinase (MMP)-2 and -9 activities were measured in replicate using gelatin zymography (Controls: *n* = ; DCE: *n* = 5). Protein lysates were prepared from ground, frozen tissue infarct samples using a non-denaturing lysis buffer. Protein concentration was determined using a standard bicinchoninic acid assay. Protein samples (20 μg) were separated on a 10 % Tris-Glycine gel with 0.1 % gelatin. Gels were incubated overnight at 37 °C in developing buffer and then stained with Coomassie Brilliant Blue R-250 for 1 h at room temperature and destained using a methanol, acetic acid, water (50:10:40) solution. Gelatinolytic bands representing pro- and active MMP-9 and MMP-2 were quantified utilizing a densitometer (BioRad GS-800, Hercules, California).

### Western blotting

Frozen myocardium samples from the core infarct zone were ground and whole cell extracts were prepared. Supernatants were collected and protein concentration was measured using the bicinchoninic acid assay. Protein samples (15–20 μg) were separated on polyacrylamide gels using a mini-vertical electrophoresis system (60–90 min, 120 V) and transblotted onto PVDF membranes, and incubated overnight at 4 °C with the required primary antibodies (Table 1). Following washes, the membranes were incubated with the required horseradish peroxidase-conjugated secondary antibodies (Table 1). Protein bands were identified by chemiluminescence (Amersham, Piscataway, NJ), followed by densitometric quantification on a BioRad densitometer.

**Table 1:** List of primary and secondary antibodies utilized for western immunoblotting experiments

Primary antibodies (Cat #)	Source	Supplier	Dilution	Application(s)
Akt (9272)	Rab PC	Cell Signaling Technol.	1:1000	WB
P-Akt (9271) (Ser473)	Rab PC	Cell Signaling Technol.	1:1000	WB
ß-Actin	Mouse MC	Sigma-Aldrich	1:5000	WB
Secondary antibodies (Cat #)	Source	Supplier	Dilution	Application(s)
Goat Anti-Mouse IgG-HRP Conjugate (170-6516)	Goat	BioRad	1:5000	WB
Anti-Rab IgG-HRP conjugate (170-6515)	Goat	BioRad	1:10000	WB

*MC* monoclonal, *PC* polyclonal, *WB* western blotting

### Statistics

Data were presented as continuous variables and expressed as mean ± SD. Categorical variables (TIMI flow grades, presence of MVO) were expressed in frequencies (%) and were compared using the Fisher’s exact test. Continuous variables (infarct size, persistent MVO size, transmurality, global/regional cardiac function, MMP levels, and Akt phosphorylation ratios) were expressed as mean ± SD and statistical significance was determined using a Student’s *t*-test. A p value <0.05 was considered statistically significant. Statistical analysis was performed by the Graphpad Prism five Software (La Jolla, CA).

## Results

### Characterization of microthrombi particles

**Fig. 2:**
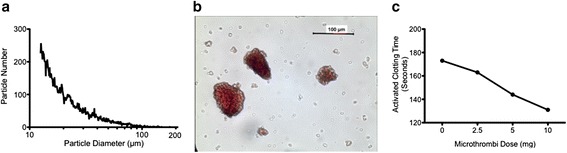
Analysis of microthrombi particles. **a**. Coulter counter assessment of particle size versus total number of a representative sample. >98 % of microthrombi particles within the sample were <100 μm in diameter. **b**. Representative light micrographic image showing several microthrombi particles (200× magnification). **c**. A representative experiment indicating a dose-related effect of microthrombi on reducing activated clotting time (ACT)

The diameter of >98 % of microthrombi particles was between 12.5 μm (aperture tube lower limit) and 100 μm as determined using the Multisizer™ III coulter counter (Fig. 2a). Light microscopy imaging confirmed this range of particle size diameter (Fig. 2b). These particles were shown to have a dose-dependent effect on lowering activated clotting time (ACT) in blood samples collected separately from three animals (Fig. 2c).

### Animal procedures

A total of 51 pigs underwent the AMI protocol, with 88 % survival. Four pigs died during the procedure due to ventricular arrhythmias and two pigs had sudden death at 1d post-AMI.

### 3 days post-AMI

#### Coronary angiography

**Fig. 3:**
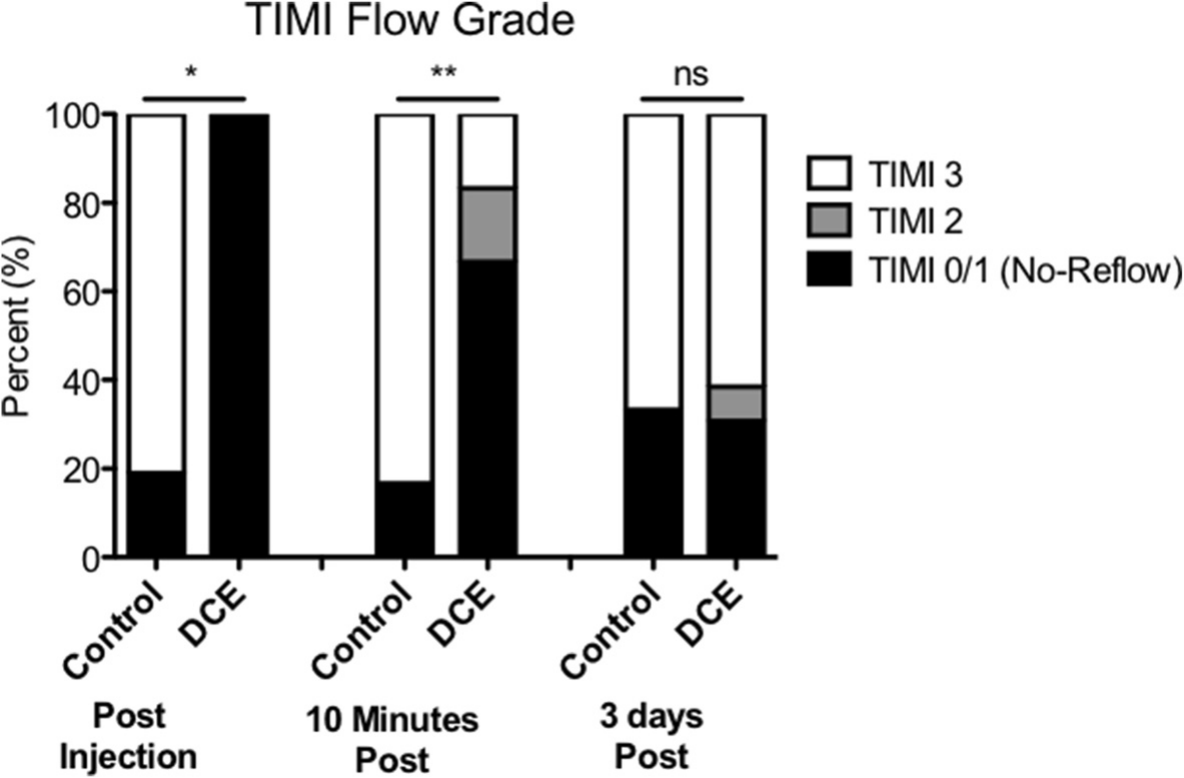
TIMI flow grades in the LAD following AMI. NR (TIMI-0 and -1 flow grades) was significantly increased immediately and at 10 min post injection of microthrombi in the DCE group. *p = 0.0001; **p = 0.006

All DCE pigs had NR immediately post DCE as mandated by protocol (Fig. 3). In contrast, only 19 % of control pigs had NR (*p* = 0.0001). At 10 min following DCE or saline injection, significant differences in TIMI flow grade between groups were still present (*p* = 0.006), although an improvement to either TIMI-2 or -3 was observed in 1/3 of the DCE animals, while the TIMI flow in control animals remained unchanged except in 1 case. At 3d post-AMI, TIMI flow grade in the DCE group had improved and there were no significant differences in TIMI flow grade between groups.

#### Serum troponin-I biomarkers of cardiac injury

There were no baseline differences in the level of troponin-I between the two groups. At 3d post-AMI, the DCE group demonstrated increased serum troponin-I levels compared to controls (13.3 ± 9.4 versus 6.9 ± 2.7 ng/uL, *p* < 0.05). There were no differences between the percentage of control and DCE animals that required defibrillation during the infarct procedure (Control: 7 of 15 animals [47 %]; DCE: 6 of 13 animals [46 %], *p* = 1.00).

#### CMR

##### Perfusion territory and infarct size

**Fig. 4:**
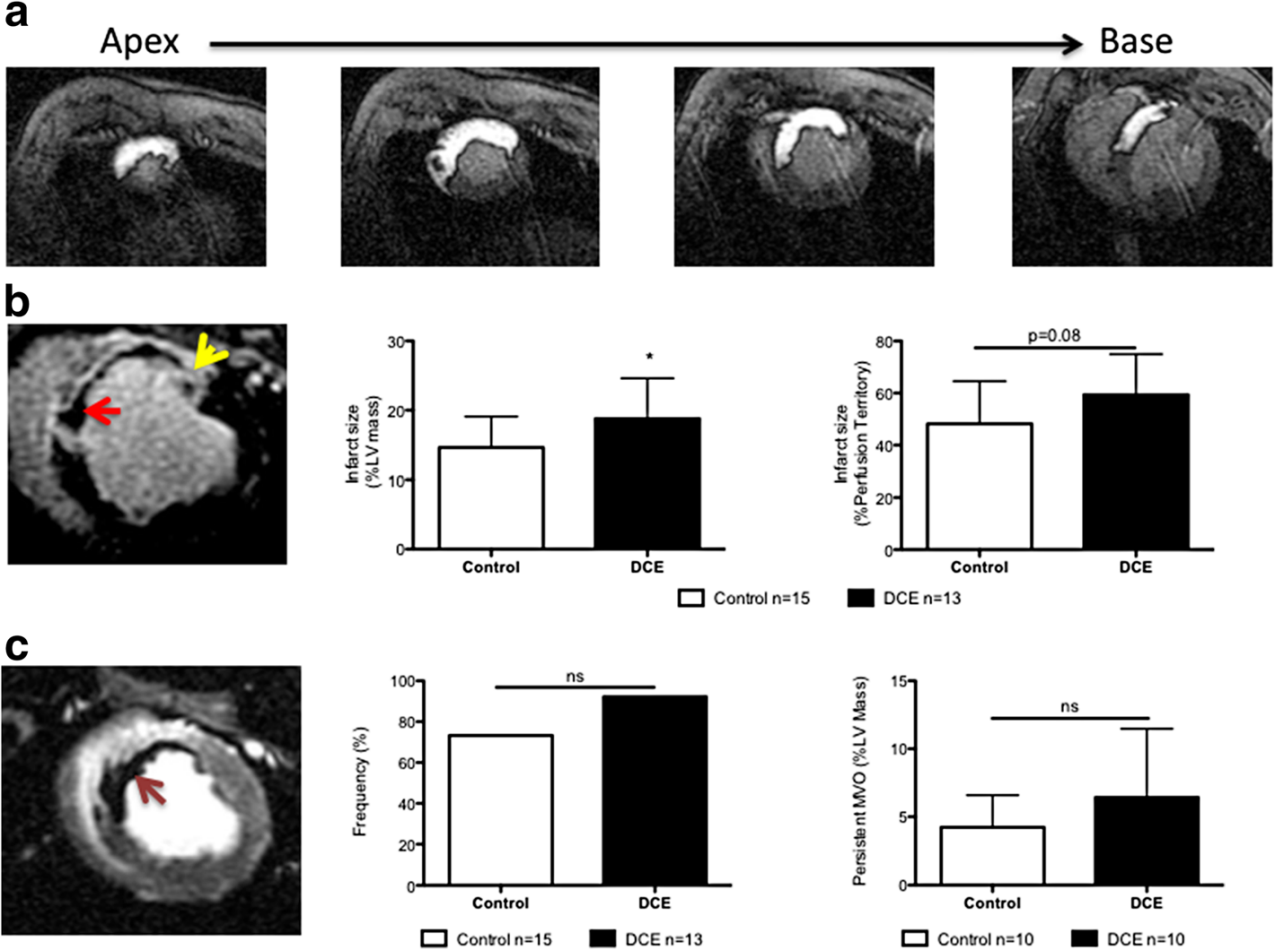
Baseline and 3d post-infarct CMR with gadolinium-DTPA. **a**. Baseline myocardial perfusion territory images (seen as white enhancement) of the mid LAD from apex to base. This territory represented 39 % of the overall left ventricular mass. **b**. LGE images showing regions of persistent MVO (red arrow) and infarct tissue (yellow arrow). Infarct size as a percent of total left ventricular mass (center) was significantly increased in DCE animals compared to controls (*p = 0.04) (Control *n* = 15; DCE *n* = 13). The DCE group also demonstrated an increased infarct size relative to the baseline perfusion territory at-risk (*p* = 0.08). **c**. EGE images depicting MVO (Left, red arrow). A non-significant increase in the proportion of animals displaying MVO (center) and persistent MVO (right) was observed in the DCE group

The myocardial perfusion territory during the baseline scan was similar between the two groups, representing 39 % of the total left ventricle in each group (Fig. 4a). There were no significant differences in the baseline perfusion territory size of control animals that had TIMI 2/3 flow versus control animals that had TIMI 0/1 flow at either 10 min or 3d post ischemia. Similar analysis with DCE animals also showed no significant differences in perfusion territory size when grouped based on TIMI flow at the two time points.

At 3D, LGE imaging showed a significant increase in infarct size in the DCE group compared to controls (18.8 ± 5.8 % versus 14.5 ± 4.4 %, *p* = 0.04) (Fig. 4b). Although infarct size as a percent of the perfusion territory was also higher in the DCE group, this did not reach statistical significance (59.2 ± 15.6 % versus 44.0 ± 16.4 %, *p* = 0.08) (Fig. 4b).

##### Microvascular obstruction (MVO)

Early gadolinium enhancement (EGE) imaging showed MVO in 12 of 13 pigs (92 %) in the DCE group compared to 11 of 15 pigs (73 %) in the control group (*p* = NS, Fig. 4c). In animals where persistent MVO was present (determined from LGE images; *n* = 10 per group), the average value as a percent of LV mass was 6.4 ± 5.0 and 4.2 ± 2.4 % in the DCE and control groups, respectively (*p* = NS, Fig. 4c).

##### Infarct transmurality

At this early time point (3d), the mean transmurality across all infarcted slices was higher in the DCE group as compared to controls (70 ± 9 % versus 60 ± 17 %, *p* = 0.06). The average transmurality of three mid-infarct myocardial slices displayed a similar trend, with increased transmurality in the DCE group compared to controls (74 ± 15 % versus 60 ± 21 %, *p* = 0.07).

##### Cardiac function

No significant differences were observed in global cardiac function parameters between groups at baseline and at 3d post-AMI (Table 2). There were also no significant differences in systolic or diastolic ventricular volumes or ejection fraction between the two groups.

**Table 2:** Cardiac function parameters assessed at baseline, 3 days, and 6 weeks post-AMI

	Baseline		3 days		6 weeks	
	Control (*n* = 15)	DCE (*n* = 13)	Control (*n* = 15)	DCE (*n* = 13)	Control (*n* = 11)	DCE (*n* = 9)
EDV (mL)	71.50 ± 12.70	76.31 ± 9.33	76.25 ± 12.30	79.79 ± 9.16	87.96 ± 12.67	93.43 ± 11.33
ESV (mL)	45.63 ± 11.36	50.34 ± 9.35	52.81 ± 13.02	53.27 ± 8.49	61.29 ± 12.23	63.53 ± 8.86
SV (mL)	25.87 ± 5.01	25.95 ± 3.34	23.45 ± 5.70	26.45 ± 3.75	26.67 ± 3.30	29.83 ± 6.89
EF (%)	36.71 ± 6.97	34.51 ± 6.10	31.27 ± 7.75	33.47 ± 5.05	30.72 ± 4.46	31.84 ± 5.66

*EDV* end diastolic volume, *EF* ejection fraction, *ESV* end systolic volume, *SV* stroke volume

### 6 weeks post-AMI

#### Coronary angiography

At 6 weeks post-AMI, all animals in both groups had TIMI ≥ 2 flow.

#### CMR imaging

##### Infarct size and transmurality

**Fig. 5:**
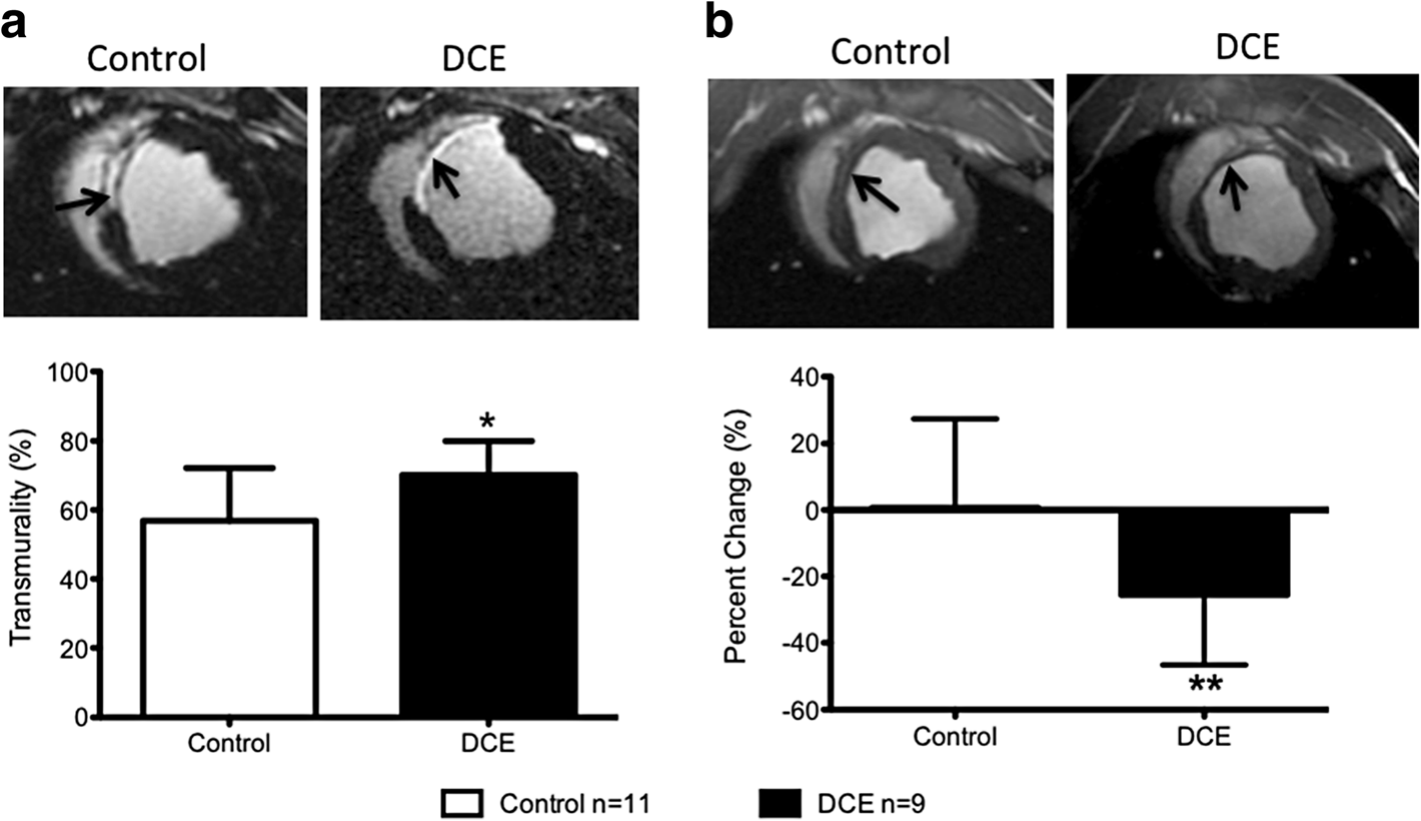
Infarct characterization 6 weeks post-AMI. **a** Top panel: Representative LGE images showing differences in infarct transmurality (infarct indicated by arrows). Lower panel: Average infarct transmurality was increased in DCE group compared to controls (*p = 0.04) (Control *n* = 11; DCE *n* = 9). **b** Top panel: Representative short axis images showing the mid-anteroseptal segment (arrows). Bottom panel: Percent change in mid anteroseptal wall thickness at 6 weeks compared to baseline in control and DCE animals. Negative values represent wall thinning. DCE treated pigs had increased wall thinning compared to controls (**p = 0.03) (Control *n* = 11; DCE *n* = 9)

At 6 weeks, infarct size in both groups had significant decreased by 50 %. There were no significant differences in infarct size (measured in grams or percent of LV mass) between the DCE group and controls (Percent of LV mass: 7.7 ± 2.4 % versus 6.0 ± 2.1 %, respectively, *p* = NS) or infarct. The average transmurality across all infarcted myocardial slices was significantly elevated in the DCE group compared with controls (70 ± 10 % versus 57 ± 15 %, *p* < 0.04, Fig. 5).

##### Cardiac function

There was more extensive ventricular thinning of the mid-anteroseptal segment in the DCE group compared to controls, based on the percent change in wall thickness at 6 weeks compared to the baseline value (-25.6 % ± 21.1 % vs. 0.7 % ± 26.8 %, *p* = 0.03, Fig. 5). Although there were significant increases in ventricular systolic and diastolic volumes from baseline to 6 weeks in both groups, no significant differences in systolic or diastolic ventricular volumes or ejection fraction were present between the two groups at 6 weeks (Table 2).

### Histology and molecular analyses

#### Histology

**Fig. 6:**
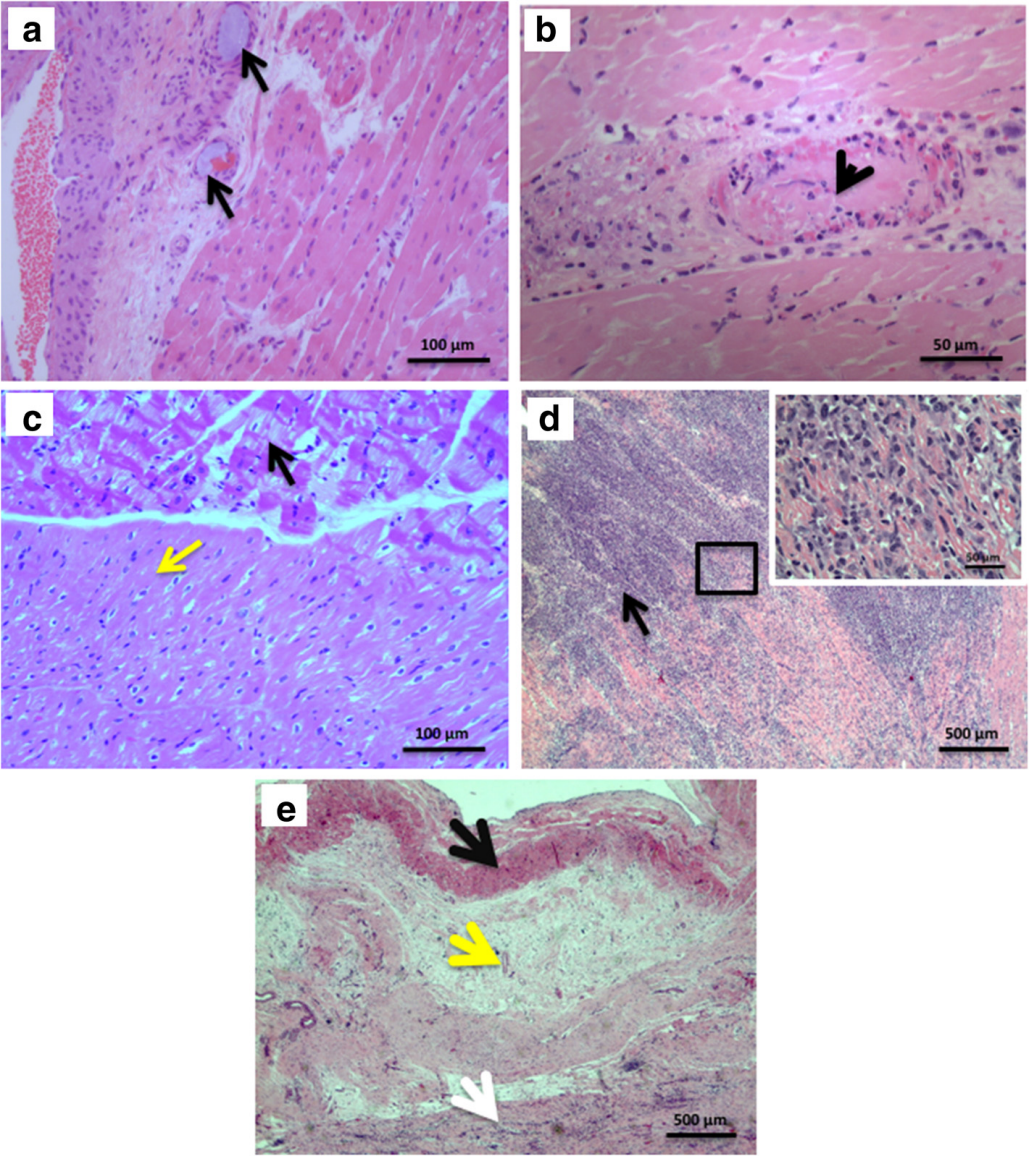
**a**-**e**. Histology of mid-infarct myocardial samples (H&E staining). **a** Microthrombi material (black arrows) present within the myocardial microvasculature at 3 h post-AMI (original magnification 200×). **b** Region of AMI showing an artery occluded by thrombus (arrow), at 3d post-AMI (original magnification 400×). **c**. Infarct junction depicting contraction band change (black arrow) and normal myocardium (yellow arrow) (original magnification 200×). **d** Extensive recruitment of inflammatory cells within the infarct zone at 3d post-AMI (arrow) (original magnification 25×). **e** Infarct zone 6 weeks post ischemia shows a subendocardial band of preserved myocytes (black arrow), and a large zone of fibrous tissue (yellow arrow) which has replaced the myocardium, and some residual inflammatory cells (white arrow) (original magnification 25×)

Tissue was studied at three time points following AMI: 3 h, 3 days and 6 weeks. There were no prominent differences between the DCE group and controls at any of the time points other than the anticipated presence of thrombi with embolized material inside intramyocardial vessels within the infarct region, at both the 3 h and 3 days time points (Fig. 6a-b). At 3 h, early markers of myocardial damage were evident in both groups, including myocyte edema, contraction band necrosis, and hypereosinophilic cells (Fig. 6c). At 3d, there was extensive infiltration of inflammatory cells, mainly neutrophils and macrophages, within the infarct zone (Fig. 6d). Epicardial and myocardial arteries and microvasculature appeared normal in the vast majority of infarcts. At 6 weeks, aneurysm formation and myocardial wall thinning was observed in both groups. Extensive myocardial fibrosis was present, with some degree of residual inflammatory cell clustering at the junction of the infarct and normal myocardium (Fig. 6e).

#### Matrix metalloproteinase (MMP)

**Fig. 7:**
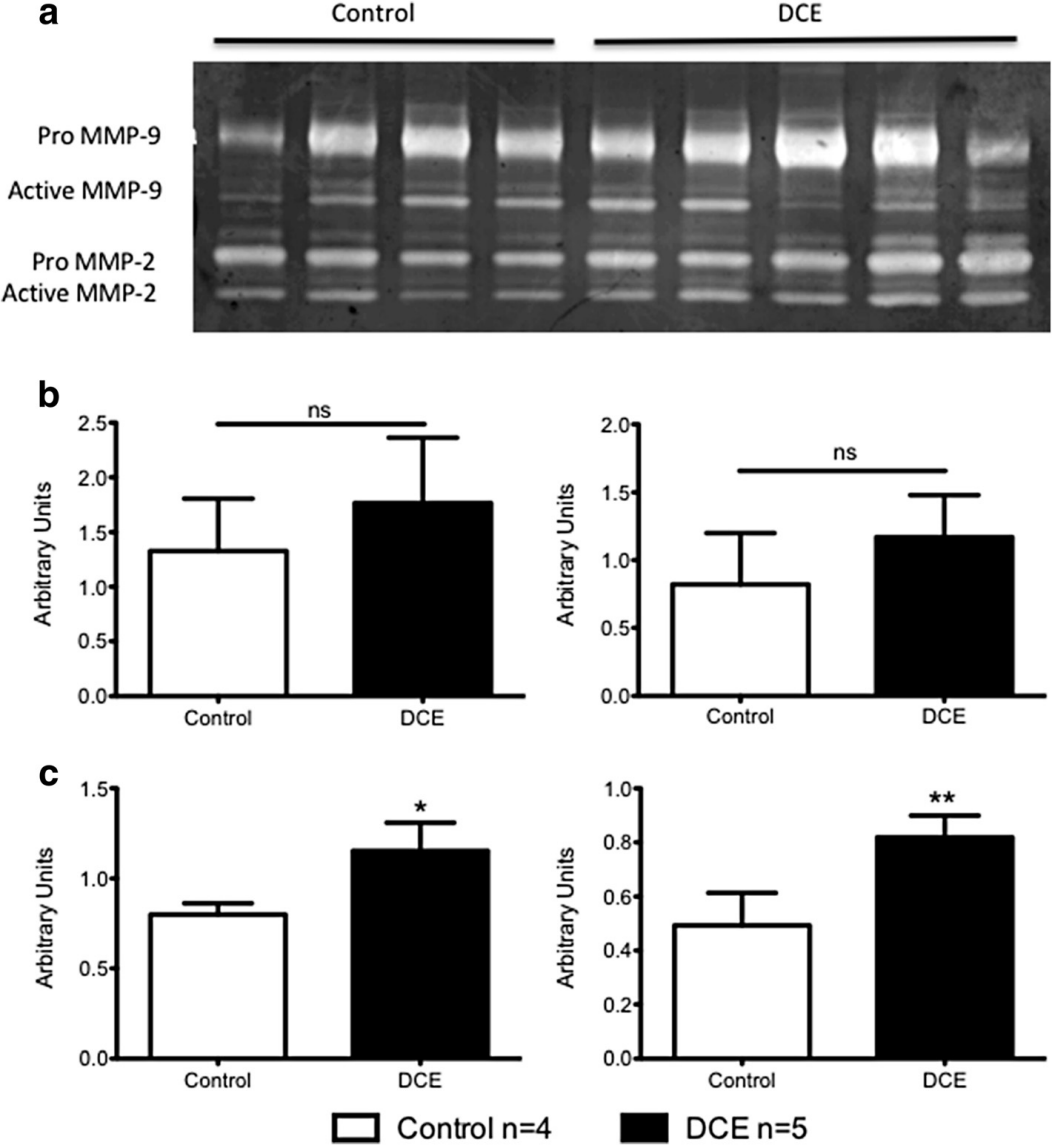
Gelatin zymography of myocardial samples at 3d post-AMI. **a**. Representative zymogram and densitometry values of pro- and active forms of MMP-9 (**b**) and MMP-2 (**c**). Pro- (*left*) and active (*right*) MMP-2 levels were significantly elevated in the DCE group compared to controls. *p = 0.004, **p = 0.002 (Control *n* = 4; DCE *n* = 5)

At 3d post-AMI, both pro- and active MMP-2 levels were elevated in the DCE group compared to controls (Pro MMP-2: 1.15 ± 0.16 versus 0.80 ± 0.06, *p* = 0.004 and Active MMP-2: 0.81 ± 0.08 versus 0.49 ± 0.12, *p* = 0.002, Fig. 7). There were no significant differences in MMP-9 levels.

#### Activation of survival kinase (Akt)

**Fig. 8:**
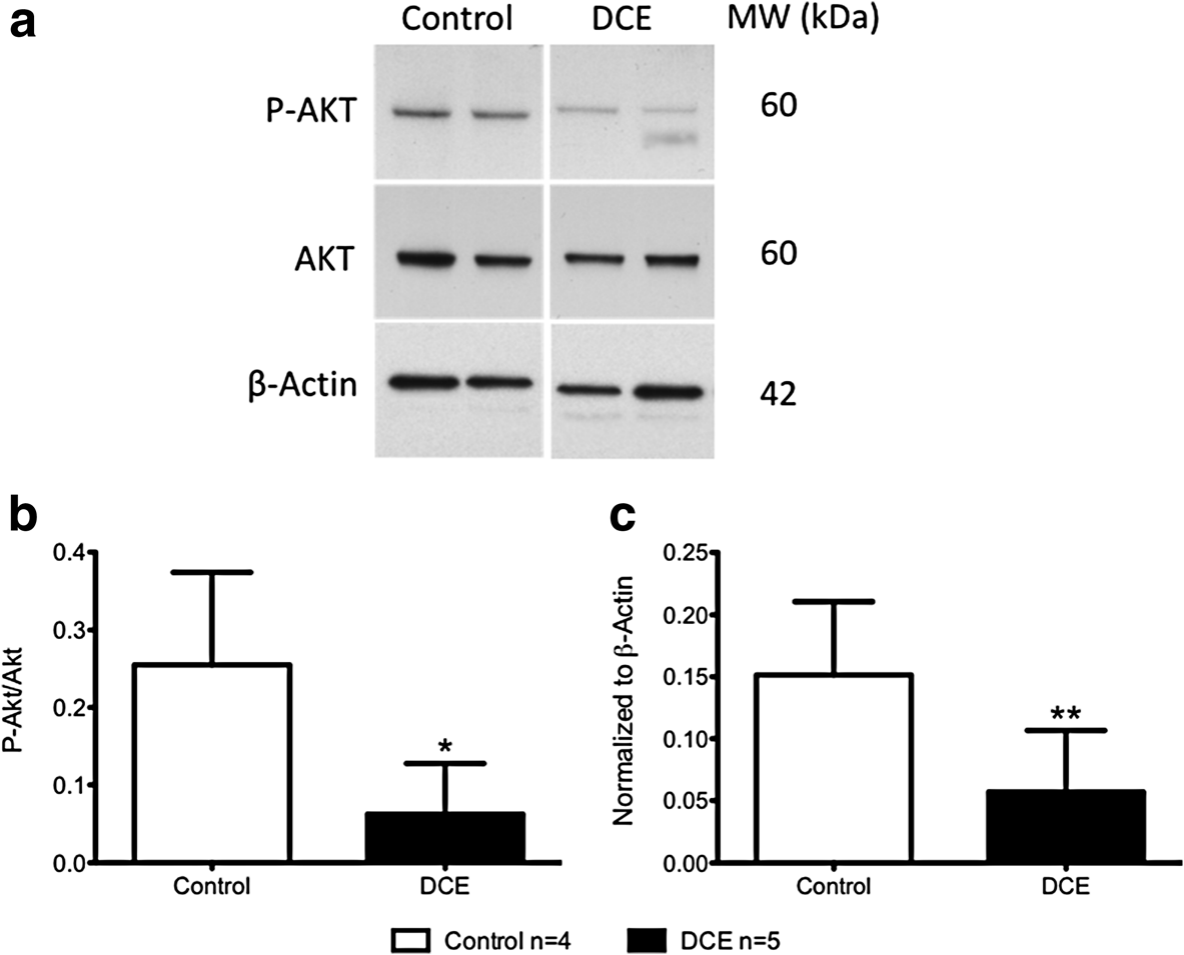
Western blotting of myocardial samples at 3d post-AMI. **a**. Representative western blots of phosphorylated Akt from infarcted myocardium of control and DCE treated animals at 3d post-AMI. Densitometry measurements of (**b**) phosphorylated Akt/total Akt ratio and (**c**) values normalized to β-actin indicated increased phosphorylated Akt in controls compared to DCE. *p = 0.02, **p = 0.03 (Control *n* = 4; DCE *n* = 5)

The ratio of phosphorylated Akt to total Akt within the infarct zone at 3d post-AMI was significantly lower in DCE group than in controls (0.06 ± 0.07 versus 0.26 ± 0.12, *p* = 0.02, Fig. 8).

## Discussion

Our study demonstrates that in a well-controlled model of moderate to severe AMI, there is an incremental effect of DCE associated with increased inflammatory markers and larger infarct size at early time points and increased ventricular wall thinning at later time points. Molecular studies indicated increased MMP-2 activity and decreased Akt phosphorylation within the infarct zone of DCE treated pigs at the early post infarct period, suggesting potential mechanisms for the adverse changes in late ventricular remodelling.

These studies were performed in a porcine model of 60 min of ischemic time prior to the embolization intervention. This ischemic time period was carefully selected to ensure the presence of a clinically relevant, patchy (ie nontransmural) infarct, which would be at least moderate in size. Previous work from our group has shown that extending the ischemic time in this model to 90 min causes transmural, large infarcts with extensive MVO that would be difficult to detect incremental damage by distal embolization. Reducing the ischemic time to 45 min produces a small or even negligible infarct without MVO that would be less representative of the infarct sizes typically encountered in clinical practice [13]. Although 60 min may seem a very short ischemic period for patients presenting with acute myocardial infarction, Hedstrom et al have previously shown large differences in the ischemic time required for 50 % infarction of area at risk between pigs and humans (37 min versus 288 min, respectively) [18]. The 14 % infarct size in our control group seems consistent with the 12.7 % infarct size documented in human studies in which the ischemic time ranges from 2.5 to 6 h, which represents the typical presentation time of most acute myocardial infarctions treated by reperfusion [19]. In contrast, the infarct size of patients presenting beyond the 6 h period was 17.9 %.

NR has been attributed to microvascular injury, due to several factors, including endothelial cell damage, myocyte swelling, inflammatory cell accumulation, reactive oxygen species and platelet-fibrin thrombi [5]. Previous experimental studies in animal models have suggested that treatment of NR may not actually affect infarct size since the microvascular damage was confined histologically to the irreversibly injured core of myocardial necrosis [5, 20]. However, these studies were not performed in an AMI model with distal embolization, which commonly occurs during PCI reperfusion therapy. In the current porcine AMI model, 80 % of pigs had normal (TIMI-3) flow grade after 60 min of ischemia alone (ie. without DCE), which still resulted in a moderate-severe sized infarct with MVO in the majority of pigs. The NR initially present in all of the DCE pigs immediately post injection did improve spontaneously over the 10-min period in 30 % of the pigs. By 3d, there were no longer differences in NR between the DCE and control groups (approximately 30 % in both groups). The presence of no-reflow in control animals at 10 min/3 days and the resolution of no-reflow in DCE animals at 10 min/3 days could not be attributed to differences in perfusion territory size within each group and time point. The spontaneous improvement in TIMI flow grades following DCE in our porcine study parallels the human setting, where NR following primary angioplasty is frequently transient and improves either spontaneously or in response to vasoactive agents during the revascularization procedure [4]. TIMI myocardial perfusion grade in patients with myocardial infarction treated with primary angioplasty has also been shown to improve over the initial few days [21]. However, even transient NR during PCI appears to adversely affect prognosis in patients, including in-hospital, 30 days and 6 months mortality [22, 23].

The DCE group had a modest but non-significant increase in persistent MVO at the early time point. In previous studies, the presence and size of MVO predicts larger infarct size, worse scar thinning and infarction expansion [24]. In the current study, there was an approximately 30 % increase in infarct size at 3d in the DCE group, indicating an initially more extensive infarct in the presence of DCE causing NR. Using CMR to determine the myocardial perfusion territory at risk, our study demonstrated that the increased infarct size at the early time point (3d) in DCE treated animals was due to a more extensive infarction within the perfusion territory at risk (59 % compared to 40 % in controls).

However, it was very interesting to note that at 6 weeks, there were no longer differences in overall infarct size between the two groups. There was a marked reduction (approximately 50 %) in overall infarct size in both groups. An AMI patient study from our group previously reported a 41 % reduction in infarct size in serial CMRs done initially at 1–48 h post AMI and again at 2–3 weeks [25]. This infarct shrinkage has been shown to be due to scar formation and infarct contraction [14]. Of note, these differences in infarct size in the current porcine study did not alter the overall ejection fractions or ventricular volumes. The accentuated infarct thinning in the DCE group would partially explain the lack of differences in infarct size between the two groups at the late time point.

The extent of infarct transmurality has been related to LV remodeling and dysfunction during healing following anterior infarction [26]. Furthermore, increased infarct transmurality measured by LGE CMR has also been identified as a risk factor for life threatening arrhythmias and cardiac death in patients with chronic myocardial infarction [27].

We have previously shown that additional effects of distal embolization include activation of inflammatory (TNF-alpha, IL-6) and thrombotic markers as a consequence of injecting inert polystyrene microspheres into porcine coronary arteries [28]. This activation occurred before the onset of NR, with progressive increase up to the point of NR. In the current study, two potential mechanisms may contribute to the remodeling changes as a result of DCE following ischemia-reperfusion injury. First, there was an increase in pro- and active MMP-2 levels within DCE samples compared to controls. Several studies have indicated that increased MMP levels occur post AMI and may adversely affect LV remodeling. In preclinical AMI models, MMP expression and activity increased following reperfusion [29]. It has also been shown that MMP-2 knockout mice have lower incidence of cardiac rupture and adverse LV remodeling [30]. Clinical studies have demonstrated an association between higher serum MMP-2 and adverse LV remodeling [31]. In our study, the increased myocardial tissue MMP-2 levels at 3d post-AMI in the DCE group may have contributed to the proteolytic milieu that resulted in enhanced LV infarct wall thinning at 6 weeks.

Secondly, our study showed reduced Akt phosphorylation in myocardial samples from the infarct core in the DCE group compared to controls. Akt is constitutively expressed within the myocardium and controls a myriad of cellular functions including cell death, differentiation and metabolism [32]. The activation (phosphorylation) of Akt has been shown to be associated with myocyte survival and reduction in infarct size in a number of pre-clinical studies [33–35]. The higher level of phosphorylated Akt in the infarct of the control group suggests that a better pro-survival tissue environment may have contributed to the observed smaller early infarct size and less late ventricular thinning seen in controls.

Many studies have described the effects of DCE, without prior AMI, on the myocardium [28, 36–38]. These studies relied on microspheres to simulate thrombotic material and demonstrated altered coronary flow, contractile dysfunction, and an inflammatory response [38]. Very few studies, however, have investigated the role of microembolization following AMI. Skyschally et al. recently demonstrated that injection of microspheres following 90 min of ischemia resulted in a significant increase in infarct size measured using triphenyl tetrazolium chloride staining [12]. Our results also illustrate an early (3d) increase in infarct size measured using CMR. Skyschally et al., however, did not report the late effects of embolization. The results from our study suggest that the differences in infarct size between groups do not persist into the chronic phase following AMI. Moreover, the use of microspheres by the previous studies may not accurately reflect the endogenous embolization material. We have utilized biologically active microthrombi derived from porcine blood which is more representative of the embolization material present clinically. Previous studies have also utilized microthrombi derived from blood [39], however, these studies have not demonstrated its effects in the context of AMI.

### Clinical relevance

Initial enthusiasm for thrombus aspiration prior to PCI in patients with AMI was based on improvement in angiographic assessment of perfusion [40] and a reduction in 1y mortality [9]. These studies formed the basis of the 2013 ACCF/AHA guidelines that provided a Class IIa indication for aspiration thrombectomy during primary PCI for AMI [8]. However, the absence of any effect of thrombus aspiration on infarct size or LV function in recent CMR studies [10] or on mortality in the randomized TASTE [11, 41] and TOTAL trial [42], have cast doubt on the utility of this intervention. Clinical distal embolization intervention studies are challenging due to patient heterogeneity related to anatomic differences (the culprit coronary artery; the level of obstruction, the thrombus burden, the period of ischemia and collateral flow) and patient characteristics and co-morbidities (age, diabetes, hypercholesterolemia, hypertensions etc.) In the controlled experimental conditions possible in our preclinical study, we did not observe significant persistent differences in infarct size at 6 weeks post infarction, and no differences in LV volumes or ejection fraction. The effects of DCE at later time periods, such as 6 months to 1 year post infarction when remodeling changes should be complete, remain to be determined in this animal model.

### Limitations

Although we have been careful to use biologically active, thrombotic material for distal embolization, we appreciate that this model does not include atherosclerotic plaque material that may also be a component of the thrombus in AMI in patients. The presence of complex plaque material or more organized thrombotic material could alter the extent and duration of microvascular injury, since this material may be more resistant to dissolution. Secondly, since we only investigated a single ischemic time parameter, our observations are specific to the case of moderate to severe AMI, given the overall infarct size and extent of MVO, even in the control group. One would need to consider different ischemic durations to probe further how the impact of DCE varies with initial infarct severity. We suspect that as the severity of the original MI increases (longer ischemic times), there would be reduced tissue left to damage by the DCE response. However, the current study does seem to be relevant for the majority of AMI patients with a high likelihood of clinical benefit by reperfusion therapy. Lastly, our porcine infarction model is limited by the absence of the complexities of human co-morbidities such as hypertension, diabetes, endothelial dysfunction and hypercholesterolemia.

## Conclusion

DCE sufficient to cause angiographic NR significantly increases early infarct size without affecting global cardiac function. However, there were no significant effects on late infarct size or global or regional cardiac function. The increases in infarct transmurality and wall thinning in the DCE group at the late time point are of uncertain clinical significance that merits further study.

## Abbreviations

ACT: Activated clotting time
AMI: Acute myocardial infarction
CMR: Cardiovascular magnetic resonance
DCE: Distal coronary embolization
EGE: Early gadolinium enhancement
H&E: Hematoxylin and eosin
LAD: Left anterior descending
LGE: Late gadolinium enhancement
MMP: Matrix metalloproteinase
MVO: Microvascular obstruction
NR: No-reflow
PCI: Percutaneous coronary intervention
TIMI: Thrombus Aspiration in Myocardial Infarction
